# A new snouted treefrog (Anura, Hylidae, *Scinax*) from fluvial islands of the Juruena River, southern Brazilian Amazonia

**DOI:** 10.1371/journal.pone.0292441

**Published:** 2024-01-31

**Authors:** Miquéias Ferrão, James Hanken, Fabrício H. Oda, Karla M. Campião, Marcos Penhacek, Samuel Anjos, Domingo J. Rodrigues

**Affiliations:** 1 Museum of Comparative Zoology, Harvard University, Cambridge, Massachusetts, United States of America; 2 Programa de Pós-Graduação em Biodiversidade Animal, Universidade Federal de Goiás, Goiânia, Goiás, Brazil; 3 Laboratório de Parasitologia Animal, Instituto de Biociências, Fundação Universidade Federal de Mato Grosso do Sul, Campo Grande, Mato Grosso do Sul, Brazil; 4 Programa de Pós-Graduação em Ecologia e Conservação, Instituto de Biociências, Fundação Universidade Federal de Mato Grosso do Sul, Campo Grande, Mato Grosso do Sul, Brazil; 5 Departamento de Zoologia, Setor de Ciências Biológicas, Universidade Federal do Paraná, Curitiba, Paraná, Brazil; 6 Programa de Pós-Graduação em Ecologia, Instituto de Biociências, Universidade Federal de Mato Grosso, Cuiabá, Mato Grosso, Brazil; 7 Programa de Pós-Graduação em Ciências Ambientais, Universidade Federal de Mato Grosso, Sinop, Mato Grosso, Brazil; 8 Acervo Biológico da Amazônia Meridional, Universidade Federal de Mato Grosso, Sinop, Mato Grosso, Brazil; University College London, UNITED KINGDOM

## Abstract

Southern Amazonia is one of the less-explored regions by anuran taxonomists. We describe a small new species of snouted treefrog, genus *Scinax*, from this region, from a fluvial archipelago in the Juruena River, state of Mato Grosso, Brazil. The description is based on external morphology of adults and tadpoles, advertisement call and molecular data. The species is phylogenetically related to other snouted treefrogs of the *Scinax cruentomma* species group and shows the most southeastern distribution in Amazonia among its close relatives. It is distinguished from congeners mainly by its larger adult body size and bilobate vocal sac that reaches the level of the pectoral fold, a reddish-brown horizontal stripe on the iris, dark melanophores or blotches on the vocal sac and the throat of females, and the uniformly brown posterior portion of the thigh. The advertisement call comprises one pulsed note emitted at regular intervals, with a duration of 189–227 ms, 30–35 pulses/note and a dominant frequency of 2,250–2,344 Hz. The type locality is suffering several environmental impacts, including illegal mining, overfishing, unsustainable agriculture, uncontrolled logging and degradation associated with the construction of new hydroelectric dams. Further study of the biology and regional distribution of the new species is required to propose mitigation measures needed for its conservation.

## Introduction

The hyline tribe Scinaxini includes 130 recognized species distributed from eastern and southern Mexico to Argentina [1]. This tribe is composed of three genera: *Ololygon* Fitzinger [2], *Julianus* Duellman, Marion & Hedges [3] and *Scinax* Wagler [4]. The latter genus contains more than 70 described species assigned to thirteen species groups, with one species unassigned to any group [5]. Currently, there are 33 species of *Scinax* in Amazonia [5–9]. Whereas several specimens of Amazonian *Scinax* housed in herpetological collections are assigned to only a few species, such as *S*. *ruber*, *S*. *garbei*, *S*. *wandae* and *S*. *cruentomma*, these taxa likely represent species complexes with many unnamed species that await taxonomic review [5, 7, 10–14].

The rate of formal description of new species of *Scinax* is highly variable over the last 20+ years and among Amazonian regions. While six new species were described between 1990 and 1993 [15–20], only two new species were named between 1994 and 2009 [21, 22]. The use of integrative taxonomy in species discovery, associated with fieldwork in remote and poorly sampled areas, facilitated the description of six new Amazonian *Scinax* between 2014 and 2018, including four from central and southwestern Amazonia [6–8, 23–25]. Yet, poorly sampled areas in Amazonia likely harbor additional candidate species awaiting formal description [7, 12, 14].

Among the poorly sampled areas in southern Amazonia, northwestern Mato Grosso (Brazil) is least explored by anuran taxonomists: only six new species of frogs were described from here in the last two decades [26–30]. Alarmingly, this part of Amazonia is strongly threatened by an “arc of deforestation” resulting from selective and clear-cut logging, expansion of livestock grazing and agriculture [31]. To make this scenario even worse, 36 dams are slated to be constructed in the Juruena and Teles Pires Basin, northwestern Mato Grosso [32]; four are already built or under construction, and feasibility studies are underway for an additional 13 [33]. Among those being planned, three mega-dams (1,200–3,509 megawatts) will be constructed in the Juruena River alone [32]. Organisms associated with the riverine environment or fluvial islands, such as the new species described in this study, will likely be affected by disruption of the natural flood pulse caused by these dams.

While sampling anurans from islands of the Juruena River, northwestern Mato Grosso, between 2019 and 2020, we found a small snouted treefrog of the *Scinax cruentomma* group whose morphology and advertisement call do not resemble those of any described species. Integration of molecular, morphological and bioacoustic data led us to identify it is a new species, which we describe here. Additionally, we highlight the need for conservation of the new species in the face of imminent anthropization of its habitat by human infrastructure development.

## Materials and methods

### Study area and sampling

**Fig 1:**
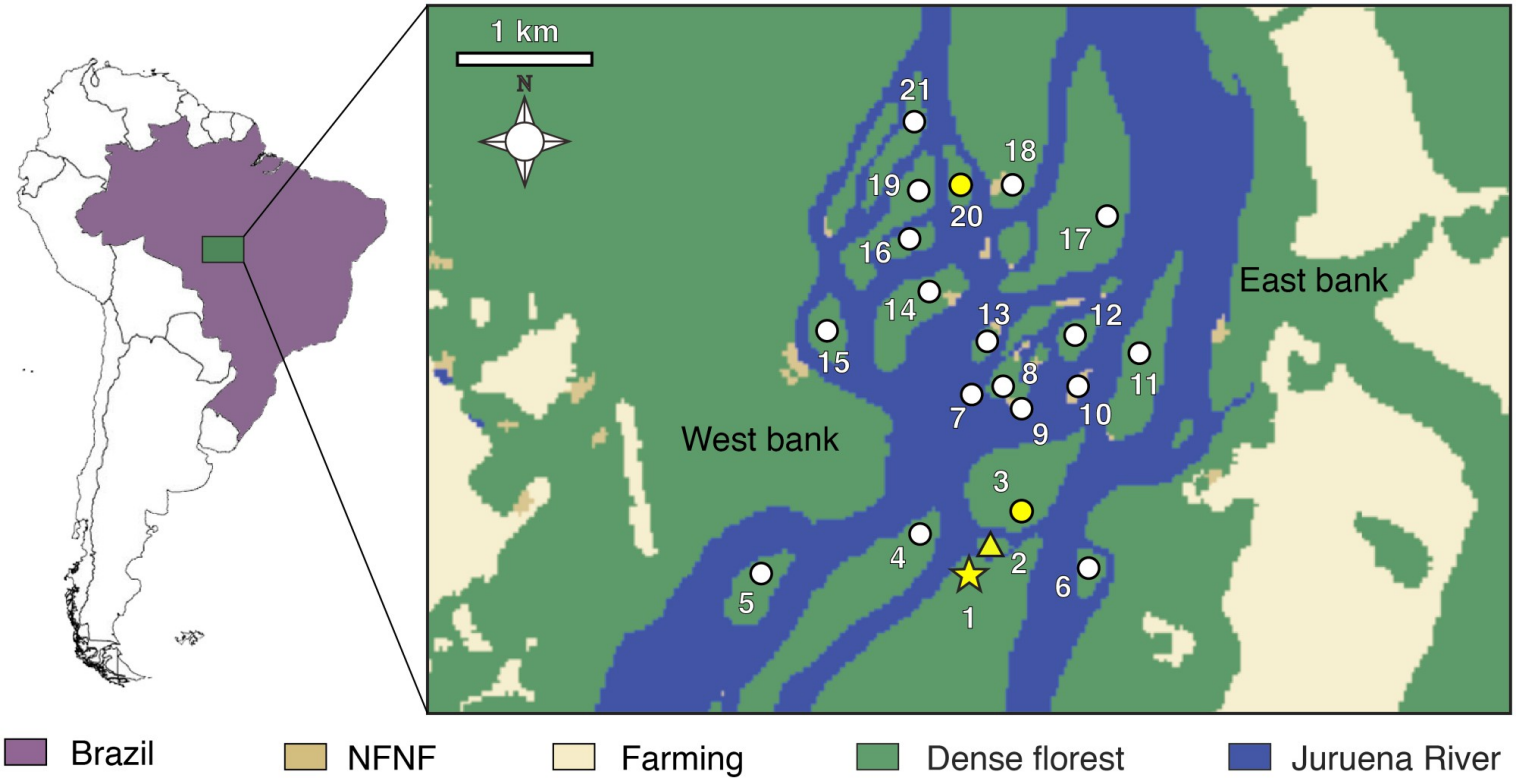
Geographic distribution of sampling sites in fluvial islands of the Juruena River, municipality of Cotriguaçu, state of Mato Grosso, Brazil. Numbers represent the sampled islands (Table 1). White and yellow symbols indicate the absence and presence of *Scinax juruena* sp. nov., respectively. Star, type locality; triangle, paratype locality. Yellow circles denote islands where uncollected individuals were recorded. NFNF, non-forest natural formation.

This study was conducted on islands in the Juruena River near São Nicolau Farm (9º48’S, 58º15’W), municipality of Cotriguaçu, northwest Mato Grosso, Brazil (Fig 1). The climate in the region is tropical with an average temperature of 24°C, relative humidity of ~ 80% and average annual rainfall of 2,034 mm. The dry season runs from April to September, the wet season from October to March [34, 35]. The Juruena is one of the main rivers in southern Amazonia; it joins the Teles Pires River to form the Tapajós River, a large tributary of the Amazon River [34]. The river’s flood elevation in the study area varies between 4 and 10 m [36] and small islands can be totally flooded, depending on their topography.

Twenty-one islands were sampled through acoustical and visual surveys (Table 1), but the new species was recorded on only four islands (Fig 1). Twelve adult specimens were collected from two islands between February 2019 and February 2020. Tadpoles were collected in February 2020 from a temporary pond where an explosive breeding event had been observed the previous month. Adults were anesthetized and killed with a topical solution of 10% benzocaine, fixed in 10% neutral-buffered formalin and preserved in 70% ethanol. Tissue samples were obtained and stored in 96% ethanol. Tadpoles were anesthetized and killed by immersion in 10% benzocaine and fixed and preserved in 5% neutral-buffered formalin. Adults, tadpoles and tissue samples are housed in the Herpetological section of the Biological Collection of Meridional Amazonia (ABAM) at the Universidade Federal de Mato Grosso, Sinop, Mato Grosso, Brazil.

**Table 1:** Geographic coordinates of sampled sites and occurrence of the new *Scinax* in islands of the Juruena River, municipality of Cotriguaçu, Mato Grosso, Brazil.

Island ID	Latitude	Longitude	Occurrence
1	9°52’5.11"S	58°12’32.02"W	Present
2	9°52’1.36"S	58°12’24.66"W	Present
3	9°51’55.40"S	58°12’19.35"W	Present
4	9°51’59.13"S	58°12’44.18"W	Absent
5	9°52’8.88"S	58°13’17.47"W	Absent
6	9°52’6.32"S	58°12’6.29"W	Absent
7	9°51’24.47"S	58°12’31.23"W	Absent
8	9°51’24.63"S	58°12’27.19"W	Absent
9	9°51’28.47"S	58°12’22.19"W	Absent
10	9°51’29.42"S	58°12’7.60"W	Absent
11	9°51’18.47"S	58°11’53.92"W	Absent
12	9°51’11.86"S	58°12’5.29"W	Absent
13	9°51’15.19"S	58°12’31.83"W	Absent
14	9°51’7.41"S	58°12’47.10"W	Absent
15	9°51’12.05"S	58°13’2.92"W	Absent
16	9°50’52.69"S	58°12’44.89"W	Absent
17	9°50’53.93"S	58°12’2.23"W	Absent
18	9°50’33.08"S	58°12’28.37"W	Absent
19	9°50’44.44"S	58°12’49.95"W	Absent
20	9°50’38.29"S	58°12’38.14"W	Present
21	9°50’23.67"S	58°12’45.73"W	Absent

Protocols of collection and animal care follow the Brazilian Federal Council for Biology resolution number 148/2012. The collection permit (Process number 30034–1) was issued by the Instituto Chico Mendes de Conservação da Biodiversidade–ICMBio (Ministry of Environment, Government of Brazil). The distribution map was made using the 2022 landcover data from MapBiomas Amazonia project, which is a multi-institutional initiative to generate annual land cover and land use maps from automatic classification processes applied to satellite imagery [37, 38]. MapBiomas data is public, open and free under a Creative Commons CC-BY-SA; the complete description of the project can be found at http://amazonia.mapbiomas.org.

The vocalization of one unvouchered male was recorded on 12 February 2019 using a CANON EOS REBEL T3 digital video camera with built-in microphone. The original MOV file was converted to WAV format with a sampling rate of 44,100 Hz and 16 bits. The video camera was sited approximately 50 cm from the recorded male. Air temperature at the time of recording is unknown. MOV and WAV files are housed in the public repository Fonoteca Neotropical Jacques Vielliard [39], University of Campinas (Campinas, Brazil).

### Phylogenetics and species delimitation

Genomic DNA was extracted from tissues of two specimens of the new species using a commercial kit (Wizard, Promega Corp., Madison, WI) following the manufacturer’s protocol. A fragment of the 16S rRNA mitochondrial gene was amplified through the polymerase chain reaction (PCR) using 16Sar (CGCCTGTTTATCAAAAACAT) and 16Sbr (GCCGTCTGAACTCAGATCGCAT) primers [40]. The PCR protocol follows [12]; PCR products were purified using ExoSAP-IT (USB Corporation, Ohio, USA). The Big Dye Terminator Cycle Sequencing Kit was used according to the manufacturer’s recommendations to sequence reactions in an automated sequencer ABI 3130xl (Applied Biosystems, Waltham, USA). Both forward and reverse primers were used in the sequencing reactions. Sequences were visually checked and edited using Geneious Prime 2023.0.2 [41]. Edited sequences ranged in length from 585 to 588 base pairs (bp).

Sequences of the mitochondrial genes cytochrome b (cyt b, n = 42), cytochrome oxidase c subunit I (COI, n = 56), 12S + intervening tRNA^Val^ and 16 rRNA (12S‑tRNA^Val^‑16S, n = 84), 3ʹ section of 16S rRNA + intervening tRNA^Leu^ (16S-tRNA^Leu^, n = 48) and NADH dehydrogenase subunit 1 + intervening tRNA^Ile^ (ND1‑tRNA^Ile^, n = 50) and of the nuclear genes chemokine receptor type 4 (CXCR4, n = 18), proopiomelanocortin (POMC, n = 42), seven in absentia homolog 1 (SIAH, n = 38), recombination activating gene 1 (RAG1, n = 51), rhodopsin exon 1 (RHOD, n = 50) and tyrosinase (TYR, n = 52) were retrieved from GenBank to better infer phylogenetic relationships of the new species. Gene sampling follows Araujo-Vieira et al. [5]. Representative sequences were retrieved from up to five specimens each of both described (n = 7) and candidate (n = 8) species of the *S*. *cruentomma* group, and from one specimen each of up to four species of the remaining 12 species groups of *Scinax* (sensu Araujo-Vieira et al. [5]). Five and four species of *Julianus* and *Ololygon* were also retrieved, respectively. *Scartyla vigilans* was used to root the tree. Mitochondrial and nuclear genes were aligned by using the strategies E-INS-i and G-large-INS-1, respectively, in the MAFFT online server with default parameters [42]. Alignments were concatenated with Concatenator [43]. The final matrix was composed of 86 terminals and 7,774 bp. GenBank accession numbers of sequences used are presented in S1 Table and were published by [5, 7, 10–12, 14, 23].

The concatenated alignment was split into 28 initial partitions, one for 12S‑tRNA^Val^‑16S, another one for 16S-tRNALeu, and one for each codon of protein-coding genes. Best-fit partitioning schemes and nucleotide models were inferred through MODELFINDER [44] implemented in IQ-TREE [45]. Phylogenetic relationships of the new species were reconstructed through Maximum Likelihood in IQ-TREE. Node support was calculated using 10,000 ultrafast bootstrap approximation replicates [46] with a maximum 10,000 iterations (unsuccessful iterations to stop > 100), and a 0.99 minimum correlation coefficient. The uncorrected pairwise p-distance and Kimura-2-parameter distance [47] between the new species and each of its close relatives were calculated in MEGA 11 [48] using the default parameters and the option *pairwise deletion*.

### Morphology

Maturity and sex of adult specimens were inferred from the presence or absence of secondary sexual characters (e.g., vocal sacs, vocal slits and nuptial excrescences on the prepollex in males; eggs in females). Morphometric measurements were taken using digital calipers accurate to 0.1 mm. Nine morphometric measurements were taken according to Duellman [49]: snout–vent length (SVL), head length (HL), head width (HW), horizontal eye diameter (ED), internarial distance (IND), interorbital distance (IOD), horizontal tympanum diameter (TD), tibia length (TL) and foot length (FL). Three additional measurements followed Napoli [50]: eye-nostril distance (END), third finger disk diameter (3FD) and fourth toe disk diameter (4TD). A final three measurements followed Heyer et al. [51]: length of tarsus (TAL), hand length (HAL) and thigh length (THL). Snout shape was categorized according to Duellman [49]. Webbing formula follows Savage & Heyer [52] as modified by Myers & Duellman [53]. Nuptial pad morphology follows Luna et al. [54], skin texture terminology follows Kok & Kalamandeen [55]. Morphometric measurements are presented in S2 Table.

Larval developmental stage of six tadpoles (lot ABAM 4101) was determined according to Gosner [56]. Description of external morphology was based on three tadpoles at stage 37. Morphometric measurements were taken with an ocular micrometer coupled to a Zeiss stereomicroscope (S3 Table). Eight measurements follow Altig & McDiarmid [57]: body length [BL], internarial distance [IND], interorbital distance [IOD], maximum tail height [MTH], tail length [TAL], tail muscle height at body-tail junction [TMH], tail muscle width at the same level as TML [TMW] and total length [TTL]); five follow Haas [58]: body height [BH], maximum body width [BW], eye diameter [ED], oral disk width [ODW] and distance of snout to center of spiracle [SS]); and two follow Randrianiaina et al. [59]: lower fin height [LFH] and upper fin height [UFH]). Coloration in life was described from photographs of recently collected tadpoles. Marginal papillae row (MPRL) and labial keratodont row (LKRF) formulae follow Schulze et al. [60]. Tadpole description format follows Schulze et al. [60].

### Bioacustics

Advertisement calls were analyzed using Raven 1.6 [61] with the following configuration: Blackman window, 3 dB; filter bandwidth, 80 Hz; overlap, 80%; hop size, 4.1 ms; and DFT size, 2,048 samples. Each call was described using the call-centered approach [62]. The following spectral and temporal parameters were measured from oscillograms and spectrograms: call duration (CD), call period (CP), call repetition rate (CR), inter-call interval (ICI), pulse duration (PD), number of pulses per call (PN), inter-pulse interval (IPI), pulse period (PP), pulse repetition rate (PRR) and dominant frequency (DF). Call repetition rate and pulse repetition rate were calculated as 1 min and 1 sec divided by the call and pulse period, respectively; dominant frequency was obtained using the peak frequency function. Call terminology follows [62]. Visual representation of the advertisement call was generated in the R environment [63] using the *Seewave* 2.0.5 [64] set up as follows: Hanning window, overlap of 85% and 256 points of resolution (FFT).

### Nomenclatural acts

The electronic edition of this article conforms to the requirements of the amended International Code of Zoological Nomenclature. Hence, the new names contained herein are available under that Code from the electronic edition of this article. This published work and the nomenclatural acts it contains have been registered in ZooBank, the online registration system for the ICZN. The ZooBank LSIDs (Life Science Identifiers) can be resolved and the associated information viewed through any standard web browser by appending the LSID to the prefix “http://zoobank.org/”. The LSID for this publication is: urn:lsid:zoobank.org:pub:B502ECB7-99B7-4998-8F80-2FAA9260BD0D. The electronic edition of this work was published in a journal with an ISSN; it has been archived and is available from the following digital repository: LOCKSS.

### Species comparison and taxonomic authorities

In their emblematic systematic revision of Scinaxini, Araujo-Vieira et al. [5] delimited thirteen species groups within *Scinax* and provided diagnostic characters for each. The new species is summarily compared to twelve of these species group and compared in detail with recognized species of the *S*. *cruentomma* group: ***S*. *albertinae*** Ferrão, Moravec, Ferreira, Moraes & Hanken, 2022 [7], ***S*. *altae*** (Dunn, 1933) [65], ***S*. *baumgardneri*** (Rivero, 1961) [66], ***S*. *blairi*** (Fouquette & Pyburn, 1972) [67], ***S*. *cruentomma*** (Duellman, 1972) [68], ***S*. *exiguus*** (Duellman, 1986) [69], ***S*. *karenanneae*** (Pyburn, 1993) [18], ***S*. *lindsayi*** Pyburn, 1992 [17], ***S*. *manriquei*** Barrio-Amorós, Orellana & Chacón-Ortiz, 2004 [70], ***S*. *staufferi*** (Cope, 1865) [71], ***S*. *strussmannae*** Ferrão, Moravec, Kaefer, Fraga & Lima, 2018 [8] and ***S*. *wandae*** (Pyburn & Fouquette, 1971) [72].

## Results

### Phylogenetic analyses

**Fig 2:**
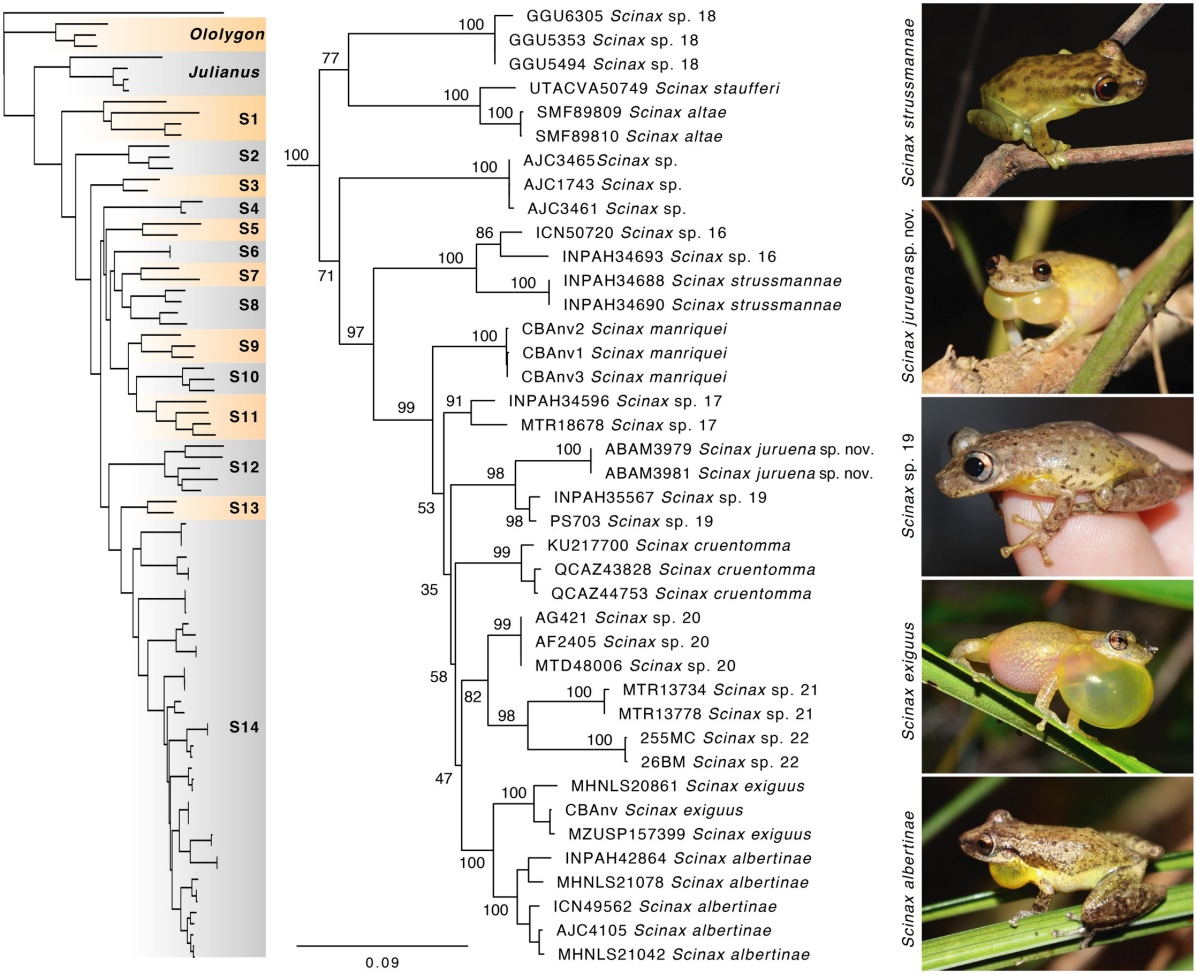
Phylogenetic relationships among *Scinax juruena* sp. nov. and closely related species of the *S*. *cruentomma* group. The phylogenetic tree was reconstructed through Maximum Likelihood (ML) based on 7,774 bp of nuclear and mitochondrial genes. Numbers near nodes are bootstrap values. The complete phylogenetic tree is illustrated in S1 Fig. Abbreviation: S1, *S*. gr. *rostratus*; S2, *S*. gr. *auratus*; S3, *S*. gr. *squalirostris*; S4, *S*. gr. *boesemani*; S5, *S*. gr. *danae*; S6, *S*. gr. *danae*; S7, *S*. *pachycrus*; S8, *S*. gr. *eurydice*; S9, *S*. gr. *granulatus*; S10, *S*. gr. *funereus*; S11, *S*. gr. *fuscovarius*; S12, *S*. gr. *nasicus*; S13, *S*. gr. *fuscomarginatus*; S14, *S*. gr. *cruentomma*.

The phylogenetic tree based on mitochondrial and nuclear genes shows the genus *Scinax* as monophyletic, as well as all species groups within it (S1 Fig). The new species nests in a major clade comprising the *Scinax cruentomma* group (Fig 2), which contains seven described (*S*. *staufferi*, *S*. *altae*, *S*. *strussmannae*, *S*. *manriquei*, *S*. *cruentomma*, *S*. *albertinae* and *S*. *exiguus*) and eight candidate species (*S*. sp. and *S*. sp. 16–22). The new species clade is strongly supported as sister to *Scinax* sp. 19, a candidate species from riverine forest in Rondônia, Pará and Amazonas states, Brazil (Fig 2). This group, in turn, is poorly supported as sister to a clade composed of *S*. *albertinae*, *S*. *exiguus*, *S*. *cruentomma* and three candidate species, *Scinax* sp. 20–22 (Fig 2).

Interspecific pairwise genetic distances between the new species and each of its close relatives of the *S*. *cruentomma* group all exceed 3% (Table 2). *Scinax* sp. 19 (sensu Araujo-Vieira et al. [5]) presents the lowest distances to the new species (K2P and p-distance = 3.0%), while *S*. *staufferi* shows the highest (K2P = 11.5%; p-distance = 10.1%). Within the major clade that includes the new species, the lowest distances are between *S*. *albertinae* and *S*. sp. 19, while the highest ones are between *S*. *juruena* sp. nov. and *S*. sp. 22 (K2P = 9.6%; p-distance = 14.7%).

**Table 2:** Interspecific pairwise genetic distances between *Scinax juruena* sp. nov. and its close relatives belonging to the S. cru.

	Taxa	1	2	3	4	5	6	7	8	9	10	11	12	13	14	15	16
1	*S*. *albertine*	**2.1**	10.0	5.0	3.8	5.7	5.9	10.2	10.1	10.2	8.0	6.2	9.0	5.7	5.7	6.5	7.6
2	*S*. *altae*	10.9	**0.0**	8.1	8.8	10.4	10.1	3.2	9.9	9.8	9.8	10.5	9.4	9.8	10.2	10.7	13.1
3	*S*. *cruentomma*	5.2	8.6	**0.2**	3.1	4.6	4.2	8.8	8.5	8.7	6.2	3.8	7.3	3.8	3.0	5.2	7.4
4	*S*. *exiguus*	4.0	9.4	3.2	**0.4**	5.0	5.1	9.4	9.0	10.4	7.4	4.7	9.2	3.4	4.6	5.7	6.6
5	*S*. *juruena* sp. nov.	6.0	11.3	4.8	5.3	**0.0**	5.4	10.6	9.6	9.6	7.1	4.3	8.3	3.0	5.8	6.9	8.8
6	*S*. *manriquei*	6.2	10.9	4.4	5.3	5.7	**0.0**	10.8	8.2	8.7	6.8	5.3	7.8	3.9	5.4	6.9	9.3
7	*S*. *staufferi*	11.0	3.3	9.4	10.1	11.5	11.7	-	10.5	9.4	9.8	10.6	9.4	10.7	11.1	11.1	13.3
8	*S*. *strussmannae*	11.0	10.7	9.1	9.8	10.5	8.8	11.5	**0.0**	10.0	4.5	9.8	9.8	8.6	12.4	11.8	14.7
9	*S*. sp.	11.2	10.5	9.4	11.3	10.4	9.3	10.1	10.8	**0.1**	9.0	9.9	9.7	9.5	10.9	10.8	13.8
10	*S*. sp. 16	8.6	10.6	6.5	7.9	7.5	7.2	10.6	4.7	9.7	**2.7**	7.9	9.3	7.2	9.2	9.8	12.5
11	*S*. sp. 17	6.6	11.4	3.9	4.9	4.4	5.5	11.6	10.7	10.7	8.5	**3.1**	8.6	3.9	4.8	7.6	8.3
12	*S*. sp. 18	9.7	10.1	7.7	10.0	8.9	8.3	10.1	10.6	9.3	10.1	9.2	**0.5**	8.2	9.3	10.0	12.6
13	*S*. sp. 19	6.0	10.5	3.9	3.5	3.0	4.1	11.6	9.2	10.2	7.7	4.0	8.7	**0.5**	4.8	6.2	7.3
14	*S*. sp.20	6.0	11.1	3.1	4.8	6.1	5.7	12.2	13.8	11.9	9.9	5.0	10.1	5.0	**0.0**	5.2	6.3
15	*S*. sp.21	6.9	11.7	5.4	6.0	7.3	7.3	12.0	13.0	11.8	10.6	8.1	10.8	6.5	5.4	**0.2**	6.6
16	*S*. sp.22	8.1	14.5	7.8	7.0	9.6	10.1	14.8	16.8	15.7	14.0	8.8	14.1	7.8	6.6	7.0	**0.2**

Each distance is based on a 602-bp fragment of the 16S rRNA mitochondrial gene and expressed as a percentage. Values below the diagonal represent Kimura-2-parameter distances; those above it are uncorrected p-distances. Numbers in bold are intraspecific p-distances. Candidate species are numbered as in Araujo-Vieira et al. [5]; *Scinax* sp. represents *S*. cf. *wandae* in that study.

## Taxonomic account

***Scinax juruena* sp. nov**. urn:lsid:zoobank.org:act:63EBD9E3-EE7F-4D6C-90BA-A9D1FD7A8FB5.

### Holotype

**Fig 3:**
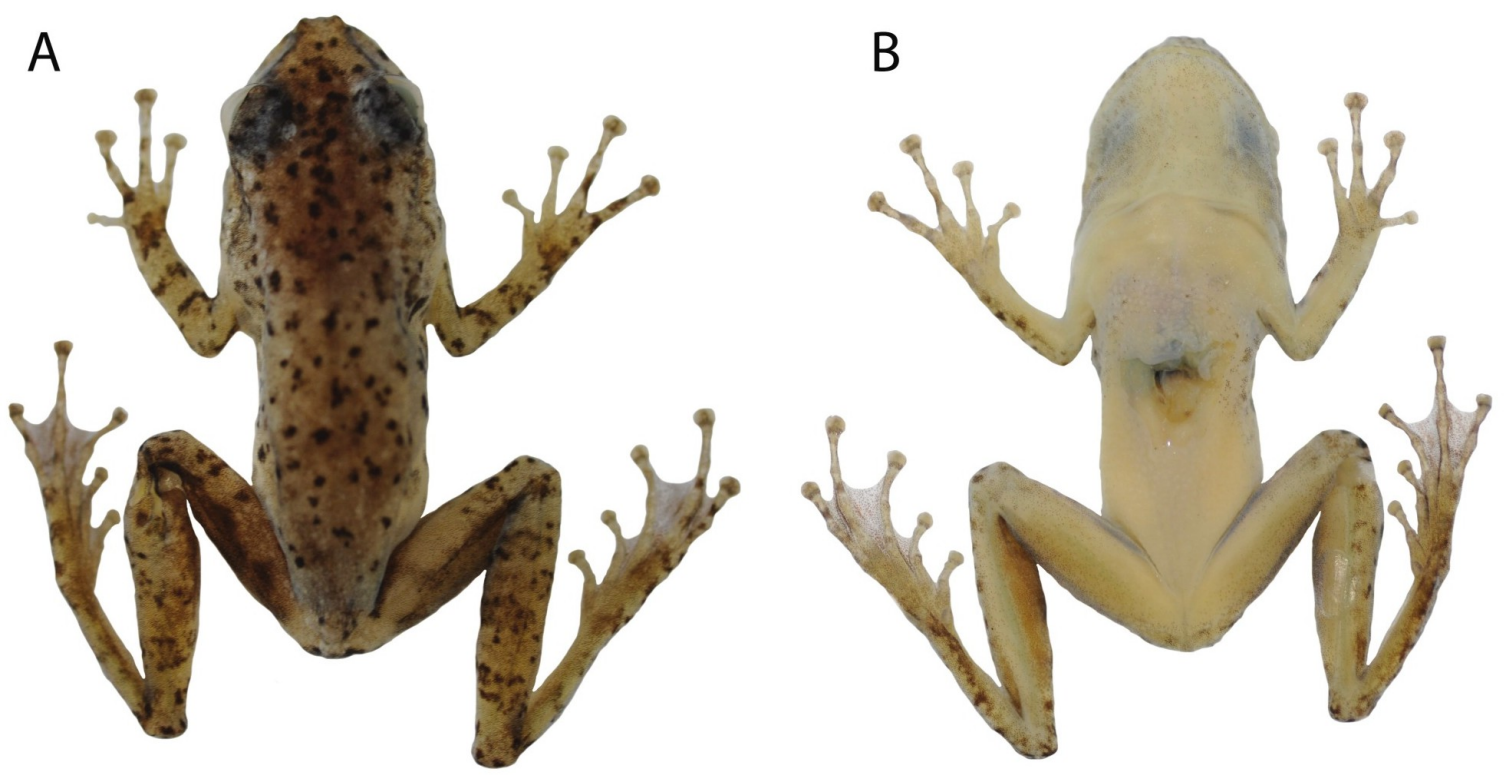
Dorsal (A) and ventral (B) views of the holotype of *Scinax juruena* sp. nov. (ABAM 3978).

ABAM 3978 (Fig 3). An adult male from an island (9°52’5.11"S, 58°12’32.02"W) in the Juruena River, municipality of Cotriguaçu, state of Mato Grosso, Brazil, collected 12 February 2019 by Domingos J. Rodrigues and Samuel Anjos.

### Paratopotypes

Eight adult specimens. Three males, ABAM 3979 (GenBank accession number MW854004), 3987 and 3988, and one female, ABAM 3980, same data as the holotype. One male, ABAM 3948, collected 1 February 2019 by Domingos J. Rodrigues and Samuel Anjos. Three males, ABAM 4088–90, collected 19 February 2020 by Marcos Penhacek and Samuel Anjos.

### Paratypes

Six adult specimens. All males, ABAM 3981 (GenBank accession number MW854005) and 3982–3986, from an island (9°52’1.36"S, 58°12’24.66"W) in the Juruena River, municipality of Cotriguaçu, state of Mato Grosso, Brazil, collected 12 February 2019 by Domingos J. Rodrigues and Samuel Anjos.

### Etymology

The specific epithet *juruena* refers to the Juruena River, where the pristine type locality of the new species is located.

*Generic placement*. The new species is assigned to the snouted treefrog genus *Scinax* based on the phylogenetic position inferred from mitochondrial and nuclear genes (Fig 2).

### Diagnosis

*Scinax juruena* sp. nov. is distinguished from congeners by the following combination of characters. SVL 24.1–27.6 mm (n = 14) in adult males; snout truncate in dorsal view; vocal sac bilobate and reaching the level of pectoral fold; dorsum covered by dark brown spots and irregularly distributed blotches, but lacking dorsal or dorsolateral dark stripes; absence of longitudinal stripe on thigh; reddish brown horizontal stripe on iris; vocal sac covered by dark melanophores or blotches; green bones. Tadpoles have LKRF 2(2)/3; upper lip unemarginated laterally; MPRF (1)/3/1; P-3 smaller than P-2 and P-1; and a dark brown horizontal bar on iris. Advertisement call is composed of one pulsed note with a call duration of 189–227 ms; a call rate of 68–89 calls/min; 30–35 pulses/note; and a dominant frequency of 2,250–2,344 Hz.

### Description of the holotype

Adult male 26.0 mm SVL (Fig 3). Body slightly slender; head slightly wider than body. Head as wide as long (HL/HW = 1.0), HL and HW each 32% of SVL. Snout truncate in dorsal view, rounded in lateral view; nostrils protuberant and elliptical, directed dorsolaterally; internarial region depressed; internarial distance 80.7% of interorbital distance. *Canthus rostralis* evident, slightly convex in dorsal view and rounded in lateral view; loreal region concave. Eyes protuberant; eye diameter 125% of IOD and 143% of IND. Interorbital region flattened, 29% of HW and 133% of UEW. Supratympanic fold barely evident, extends from posterior corner of eye to posterior portion of tympanum. Tympanum small and rounded; diameter 46% of ED; tympanum-eye distance approximately equal to TD; tympanic annulus evident and rounded, upper portion hidden by supratympanic fold. Vocal slits present, extend from anterolateral base of tongue to commissure of the jaw; tongue long and lanceolate. Dentigerous processes of vomers present, triangular and separated from each other by half of their length; four teeth on the right side, three on the left. Choanae oval. Vocal sac distinct, bilobate and subgular; when deflated, extends laterally to the area above the forearm insertion.

Upper arm as slender as forearm. Axillary membrane absent. Ulnar tubercles absent. Hand length 26.1% of SVL; fingers slender and long, poorly fringed; relative length of fingers I < II < IV < III. Finger discs large and elliptical, third finger disc diameter 36.6% of ED and 78.6% of TD; finger disc on finger I smaller than on fingers II–IV; finger webbing vestigial. Palmar tubercle bifid and flattened; thenar tubercle flattened and elliptical; distal subarticular tubercle conical on fingers I and II, rounded on fingers III and IV; supernumerary tubercles present and subconical on fingers I and II, indistinct on the others. Nuptial pad light-colored, longer than wide, extends from anterolateral area of distal subarticular tubercle on finger I to posterolateral region of thenar tubercle.

Hind limbs robust; thigh more robust than tibia. Tibia slightly longer than thigh (TL/THL = 1.06); tibia length 48.5% of SVL; thigh length 45% of SVL. Foot longer than tarsus (FT/TAL = 1.54), 83.3% of TL and 88.9% of THL. Toe discs elliptical; fourth toe disc diameter (4TD) 30% of ED and 64.3% of TD. Toes slender and long, poorly fringed; relative length of toes I < II < V < IV < III; webbing formula I 2^+^–2^1/2^ II 1^1/2^–2^1/3^ III 1^+^–2 IV 2–1^+^ V. Inner metatarsal tubercle oval and flat; outer metatarsal tubercle rounded and flat, four times smaller than inner metatarsal tubercle; subarticular tubercles single and rounded on toes II–V, subconical on toe I. Supernumerary tubercles indistinct. Tarsal fold and tubercles absent. Tubercles on heels absent. Anal opening directed dorsoventrally at the midlevel of thighs, covered by a short and slender anal flap.

In preservative, skin smooth on head, upper eyelid, dorsum and dorsal surfaces of limbs. Flanks finely granulate. Small and rounded tubercles on posterior portion of tympanum. Skin smooth on vocal sac, ventral surfaces of upper arm and forearm, throat, chest and ventral surfaces of tibia and tarsus. Skin areolate on belly and ventral surface of thighs. Small and rounded tubercles in perianal region. In life, skin texture is similar to that in preservative, except for the presence of small and irregularly distributed tubercles on dorsum and flanks in life.

In preservative (Fig 3), anterior portion of head light grey and posterior portion light brown; dorsum and upper eyelid light grey; brown canthal stripe extends from the nostril to the anterosuperior portion of tympanum; upper lip light cream; dorsal surfaces of hand, forearm, upper arm, foot, tarsus and tibia light cream; anterior surface of thigh dark brown close to the groin and light cream close to the knee, superior surface light cream, posterior surface brown. Small dark brown spots and irregularly distributed blotches on head, dorsum, flanks, arm, anterior and superior portion of thigh, foot and tarsus. Dark vertebral and lateral lines on dorsum absent. White tubercles on posterior portion of tympanum. Three inconspicuous light brown stripes on tibia, two on forearm and one on the proximal portion of hand. Anal flap dark brown; perianal tubercles white. Vocal sac light cream with several small dark and irregularly distributed melanophores; chest, belly and ventral surfaces of limbs cream. Small dark and irregularly distributed melanophores on ventral surfaces of hands, feet and hind limbs. Color in life of the holotype is unknown.

### Coloration in life of paratypes

**Fig 4:**
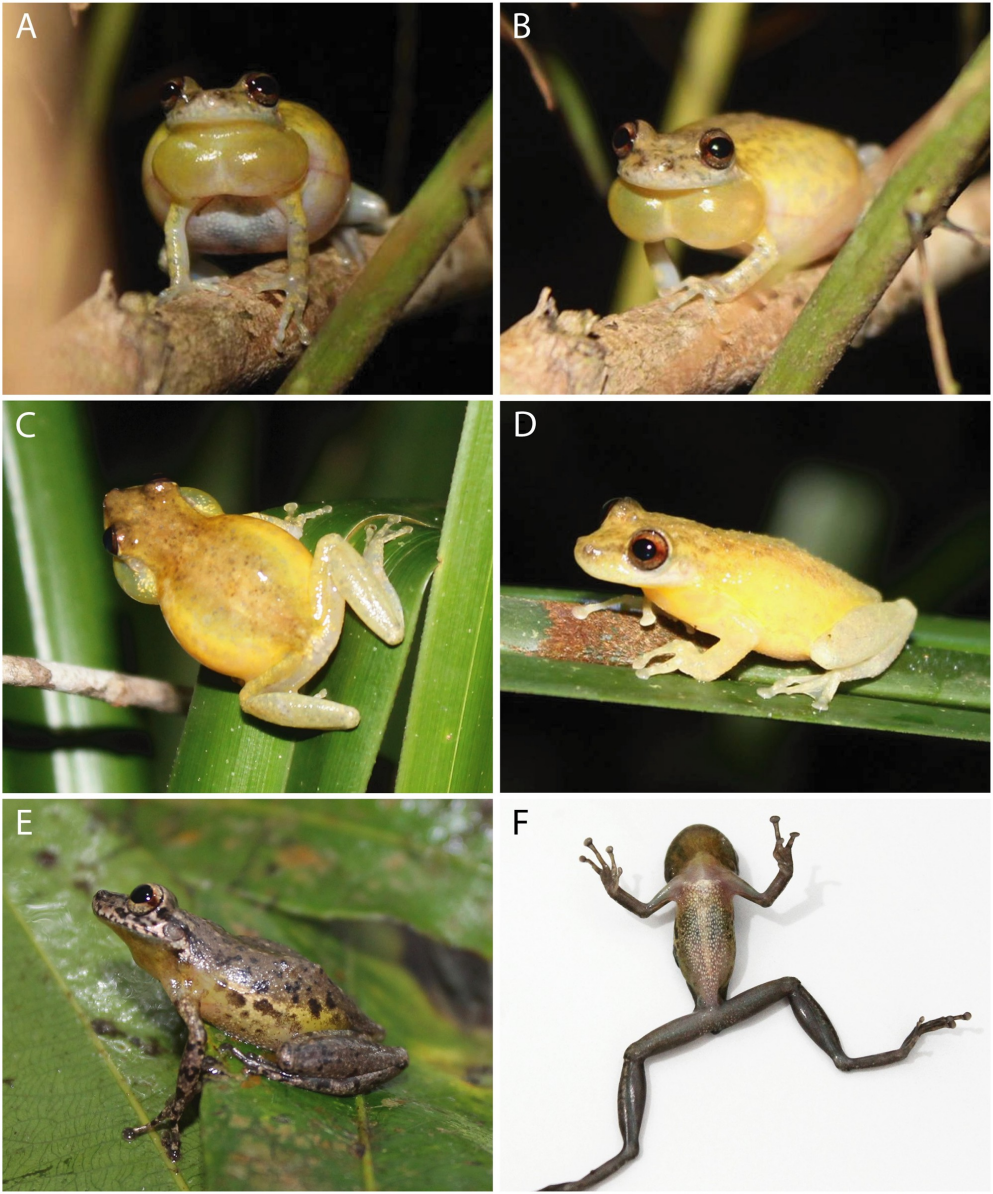
Coloration in life of *Scinax juruena* sp. nov. from islands in the Juruena River, municipality of Cotriguaçu, state of Mato Grosso, Southern Amazonia, Brazil. Nocturnal (A–D) and diurnal (E, F) color patterns.

*Scinax juruena* sp. nov. changes external coloration between night and day. At night (Fig 4A–4D), dorsal surfaces of the head and body are brownish yellow with tiny light brown dots and irregularly distributed spots; the canthal stripe is brown; upper lips are whitish grey with small grey dots; an inconspicuous light brown stripe extends from the posterior corner of the eyes to the posterior portion of the tympanum; and white tubercles cover the posterior portion of the tympanum and the lateroposterior portion of vocal sac (Fig 4C). The iris is silvery brown with a reddish brown horizontal stripe medially. Upper flanks are yellow and lower flanks pale yellow; both are covered with whitish grey spots and blotches. Dorsal surfaces of upper and lower arms are yellow and pale yellow, respectively, and covered by grey bands. The dorsal surface of the thigh is pale yellow with light grey bands; anterior and posterior surfaces are translucent. Dorsal surfaces of the tibia, tarsus and foot are tan with light gray dots and bars. Ventral surfaces of fore- and hind limbs are whitish grey. The chest and belly are pinkish white to white. In males, each lobe of the bilobate vocal sac is yellow with dark melanophores; the contact zone between them is whitish yellow (Fig 4A and 4B). In females, the throat is translucent with dark melanophores.

During the day (Fig 4E and 4F), dorsal surfaces of the head, body, upper flanks and fore- and hind limbs become greyish brown; dots, spots, blotches and bars on dorsal surfaces became darker and larger; ventral surfaces of the tarsus and foot became dark grey; and upper lips become brownish white. Lower flanks are yellowish cream and the groin is yellow; both are covered by dark brown spots and blotches. The thigh is brown both anteriorly and posteriorly; ventral surfaces of the thigh and tibia are brownish grey. The chest is translucent, the belly brownish white. In males, the vocal sac is brownish yellow with dark melanophores and small brown blotches.

### Variation

**Fig 5:**
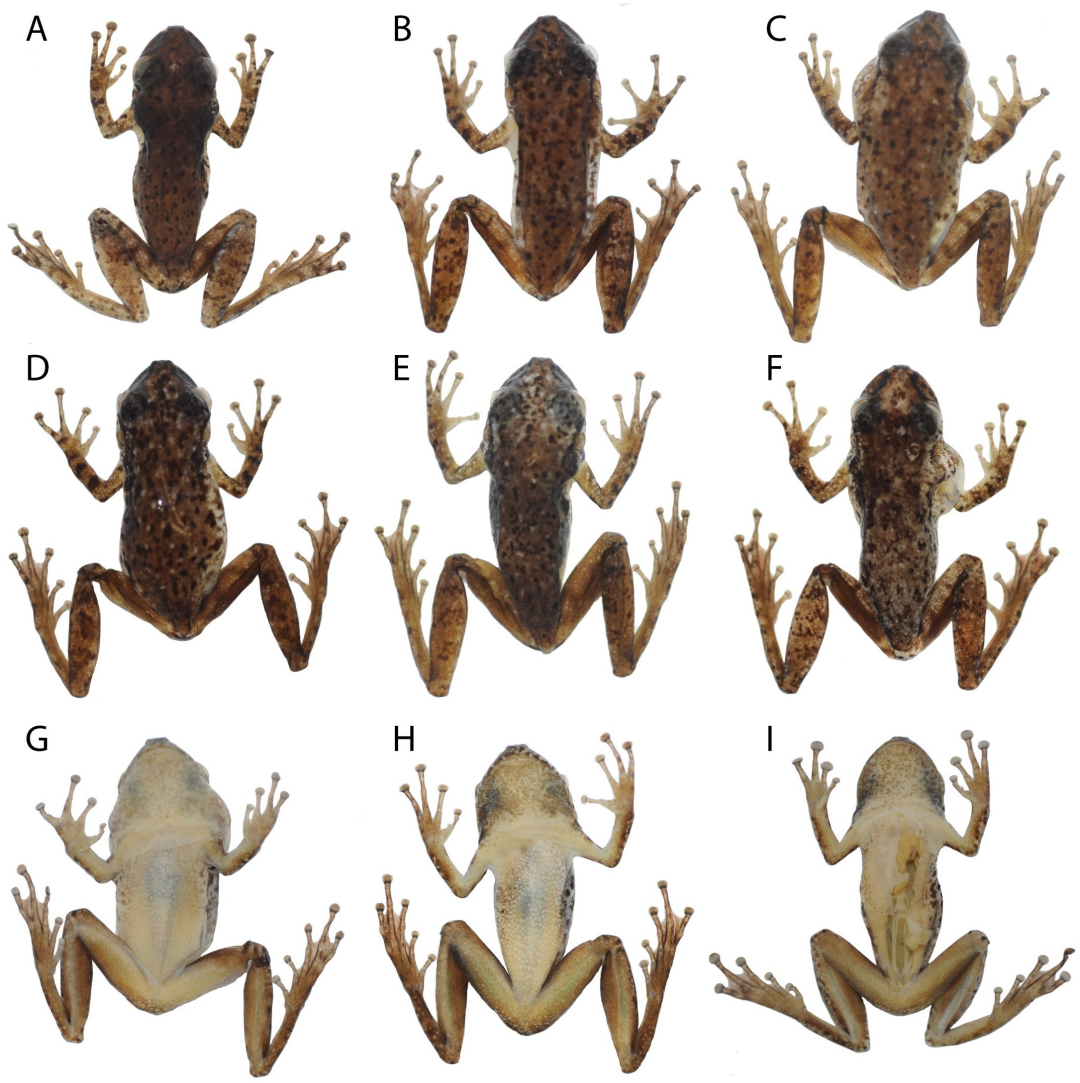
Variation of dorsal and ventral coloration in preservative of adult *Scinax juruena* sp. nov. (A) ABAM 3948, male, SVL 26.9 mm; (B) ABAM 3980, female, SVL 26.3 mm; (C) ABAM 3985, male, SVL 24.3 mm; (D) ABAM 3982, male, SVL 24.2 mm; (E) ABAM 3988, male, SVL 25.1 mm; (F) ABAM 3979, male, SVL 27.6 mm; (G) ABAM 3985; (H) ABAM 3988; (I) ABAM 3948.

External morphology of paratypes and paratopotypes of *Scinax juruena* sp. nov. largely agrees with that of the holotype; see Table 3 for measurements. The SVL of the only female falls within the range of males. Differences relate mainly to color pattern in preservative (Fig 5). Small light blotches are present in 55% of the paratypes. Whereas all paratypes have dark brown spots of varying size on the flanks, 82% of them also have elongate dark brown blotches. Brown bars on the tibia are conspicuous in 55% of the paratypes but inconspicuous in the others. Brown bars on the thigh, which may be conspicuous or inconspicuous, are present in 91% of the type series; 2–4 bars are present on each thigh. The posterior surface of the thigh is brown in 91% of the paratypes and light brown in one individual. The lateral surface of the belly is immaculate in one paratype but covered by small to medium-sized dark spots and blotches in all others (Fig 5). While the vocal sacs of all males have dark melanophores, only 20% of them show the same pattern as the holotype. An additional 20% have more pigmentation, and the remaining 60% have dark brown blotches in addition to dark melanophores. The throat of the lone female has dark melanophores arrayed as in the holotype.

**Table 3:** Morphometric measurements of *Scinax juruena* sp. nov.

Measurements	Holotype	Males (n = 14*)	Female (n = 1)
SVL	26.0	25.6 ± 1.1 (24.1–27.6)	26.3
HL	8.3	8.4 ± 0.3 (8.0–8.8)	8.8
HW	8.3	8.4 ± 0.3 (8.0–8.8)	8.8
ED	3.0	2.9 ± 0.1 (2.6–3.1)	3.0
TD	1.4	1.5 ± 0.1 (1.3–1.6)	1.5
UEW	1.8	2.0 ± 0.1 (1.7–2.1)	2.2
IOD	2.4	2.4 ± 0.2 (1.9–2.8)	2.4
IND	2.1	1.9 ± 0.1 (1.7–2.2)	2.1
END	2.7	2.8 ± 0.1 (2.6–3.1)	2.9
HAL	6.8	6.8 ± 0.3 (6.2–7.4)	7.5
3FD	1.1	1.1 ± 0.1 (0.9–1.2)	0.9
4TD	0.9	1.0 ± 0.1 (0.9–1.2)	0.9
TAL	6.8	6.8 ± 0.3 (6.3–7.2)	7.2
FL	10.5	10.4 ± 0.5 (9.4–11.1)	10.2
TL	12.6	12.1 ± 0.4 (11.4–12.7)	12.6
THL	11.8	11.5 ± 0.4 (10.7–12.0)	12.1

*, Includes the holotype. Values denote mean ± standard deviation and range (minimum–maximum). Measurement abbreviations are explained in the text.

### Advertisement call

**Fig 6:**
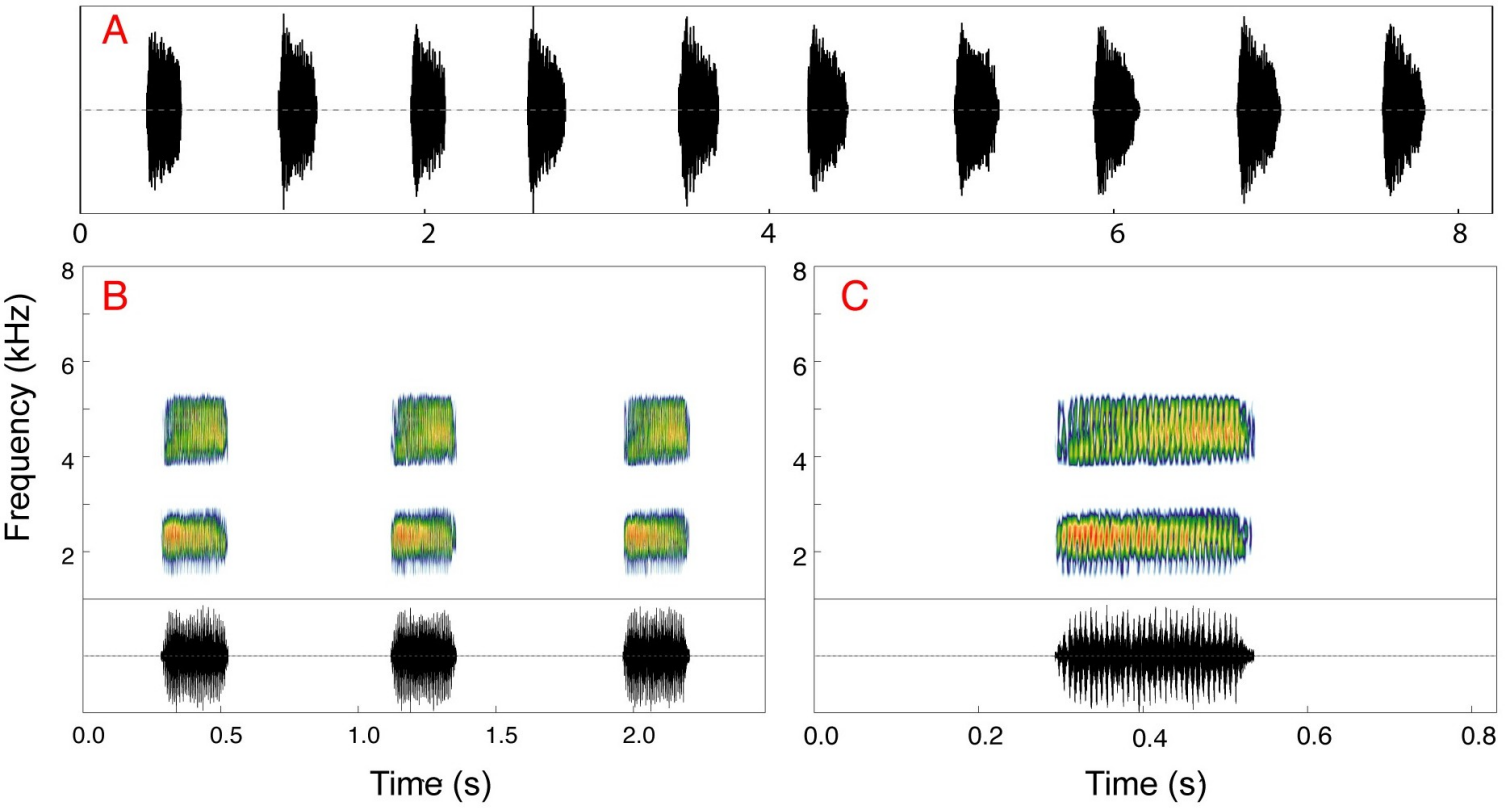
Advertisement calls of *Scinax juruena* sp. nov. from the type locality. (A) Oscillogram showing the repeated emission of 10 calls. (B) Spectrograms and (C) oscillograms of three and one calls, respectively.

The advertisement call of *Scinax juruena* sp. nov. consists of one pulsed note emitted in series (Fig 6A–6C). Each call has an average duration of 211 ms ± 15 (189–227 ms; n = 10) and an average period of 796 ms ± 67 (677–886 ms; n = 9), with an average inter-call interval of 587 ms ± 58 (488–678 ms; n = 9). Calls are emitted with an average repetition rate of 76 ± 7 calls/min (68–89 calls/min). Each note comprises 30–35 pulses (33 ± 2). Pulses have a duration of 4.5 ± 0.5 ms (4–5 ms) and a period of 6–7 ms (6.5 ± 0.5), with an inter-pulse interval of 1–3 ms (1.9 ± 0.4). Pulse repetition rate is 143–167 pulses/s (156 ± 12). Calls have two emphasized frequency bands; the dominant frequency 2,250–2,344 Hz (2,318 ± 28) corresponds to the lower band in all analyzed calls.

### Tadpoles

**Fig 7:**
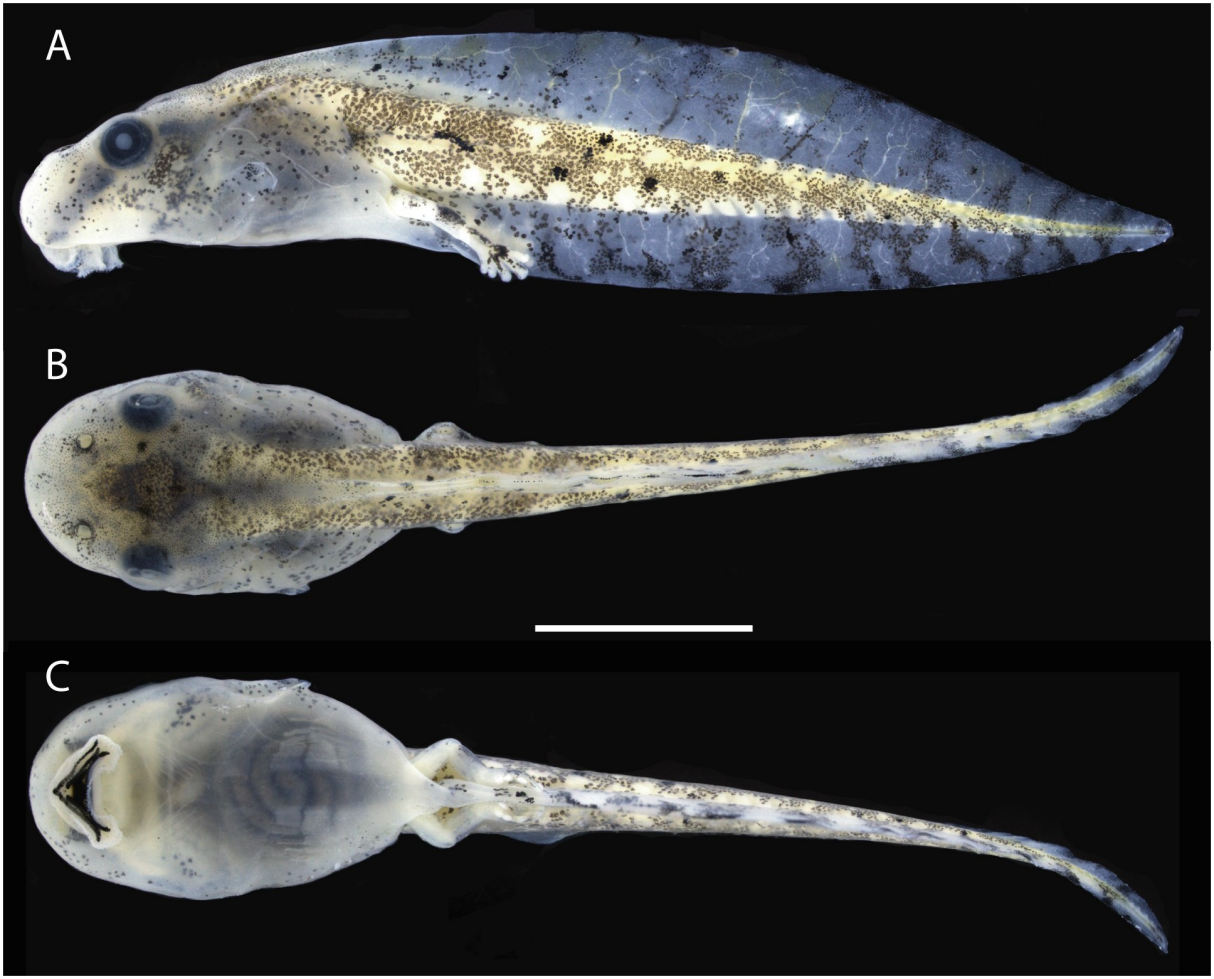
Lateral, dorsal and ventral views of a preserved tadpole of *Scinax juruena* sp. nov. Lot ABAM 4101, Gosner stage 37. Scale, 5 mm.

The following description is based in three tadpoles at Gosner stage 37 (ABAM 4101). Individual measurements are presented in Table 4. Total length 25.5 ± 2 mm (23.2–27.0 mm). Body triangular and ovoid in lateral and dorsal views, respectively (Fig 7); body length 30% ± 2 (29–32%) of total length; body longer than high (BL/BH = 2.0 ± 0.2, 1.8–2.1) and wider than high (BW/BH = 1.3 ± 0.1, 1.2–1.4). Snout rounded in dorsal and lateral views. Nostrils rounded, dorsally positioned and directed; internarial region concave; internarial distance 68% ± 4 (63–72%) of interorbital distance and 47% ± 1 (46–48%) of body width. Eyes positioned dorsolaterally; eye diameter 16% ± 1 (16–17%) of body length; interorbital region concave, interorbital distance 70% ± 6 (64–76%) of body length. Spiracle sinistral, opening directed posterodorsally, inner wall free from body. Tail longer than body (TAL/BL = 2.3 ± 0.2, 2.1–2.5), without flagellum; tail length 70% ± 2 (68–71%) of total length; maximum tail height exceeds body height (MTH/BH = 1.3 ± 0.1, 1.3–1.4); lower fin height 77% ± 1 (76–78%) of upper fin height; tail muscle width 88% ± 7 (82–95%) of its height; tail muscle height 41% ± 1 (40–42%) of maximum tail height and 28% ± 2 (25–28%) of body height.

**Table 4:** Morphometric measurements of tadpoles of *Scinax juruena* sp. nov. (lot ABAM 4101).

Trait	Gosner stage					
	36	36	36	37	37	37
BH	3.8	4.0	3.5	3.5	4.2	**4.2**
BL	7.3	7.2	6.9	7.5	8.0	**7.7**
BW	4.7	5.0	4.4	5.0	5.0	**5.0**
ED	1.2	1.2	1.1	1.3	1.3	**1.2**
IND	2.4	2.3	2.1	2.4	2.3	**2.4**
IOD	3.3	3.7	3.1	3.5	3.2	**3.8**
LFH	1.5	1.4	1.3	1.3	1.6	**1.8**
MTH	5.0	4.9	4.1	4.5	5.3	**5.8**
ODW	2.7	2.7	2.4	2.7	2.8	**2.7**
SS	5.6	5.7	5.6	6.0	6.2	**5.8**
TAL	14.6	17.0	14.8	15.7	18.2	**19.3**
TMH	2.1	2.0	2.0	1.9	2.2	**2.3**
TMW	1.8	1.8	1.5	1.8	1.8	**2.0**
TTL	21.9	24.2	21.7	23.2	26.2	**27.0**
UFH	1.9	1.8	1.6	1.7	2.1	**2.3**

Bold numbers denote the individual depicted in Fig 7.

Measurement abbreviations are explained in the text.

**Fig 8:**
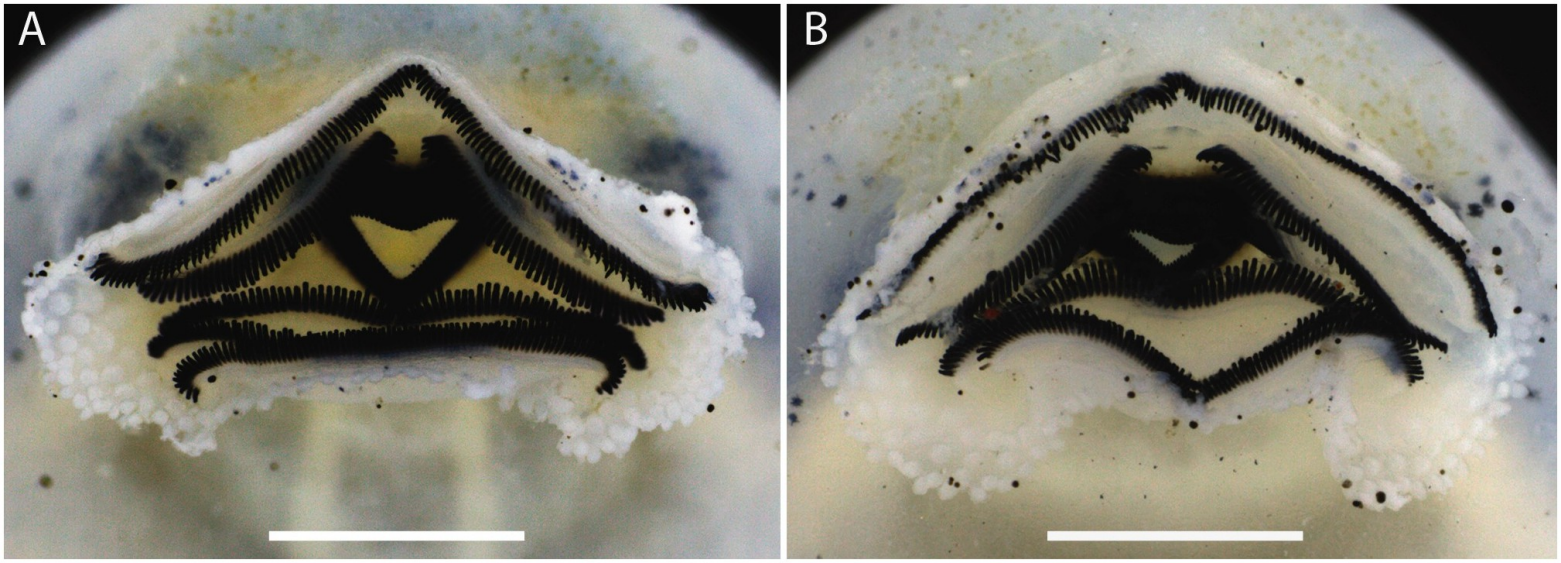
Oral disc of two preserved tadpoles of *Scinax juruena* sp. nov. Lot ABAM 4101, Gosner stage 37. Scales, 1 mm.

Oral disc located and directed anteroventrally (Figs 7A and 8), protuberant when closed. Upper lip not emarginated laterally; lower lip emarginated laterally (Fig 8). Papillae rounded and long; marginal papillae in a uniseriate row on the upper lip, with a moderate gap anteriorly, and on the central portion of the lower lip; marginal papillae in a triseriate row on the fold; marginal papillae row formula (1)/3/1. Submarginal papillae absent. Jaw sheaths serrated with conical cusps; upper jaw sheath M-shaped; lower jaw sheath V-shaped; upper jaw sheath more massive than lower jaw sheath. Labial keratodont row formula 2(2)/3; A-1 and A-2 nearly the same length, narrow gap in A-2; P-1 and P-2 approximately the same length, P-3 smaller than P-2 and P-1.

**Fig 9:**
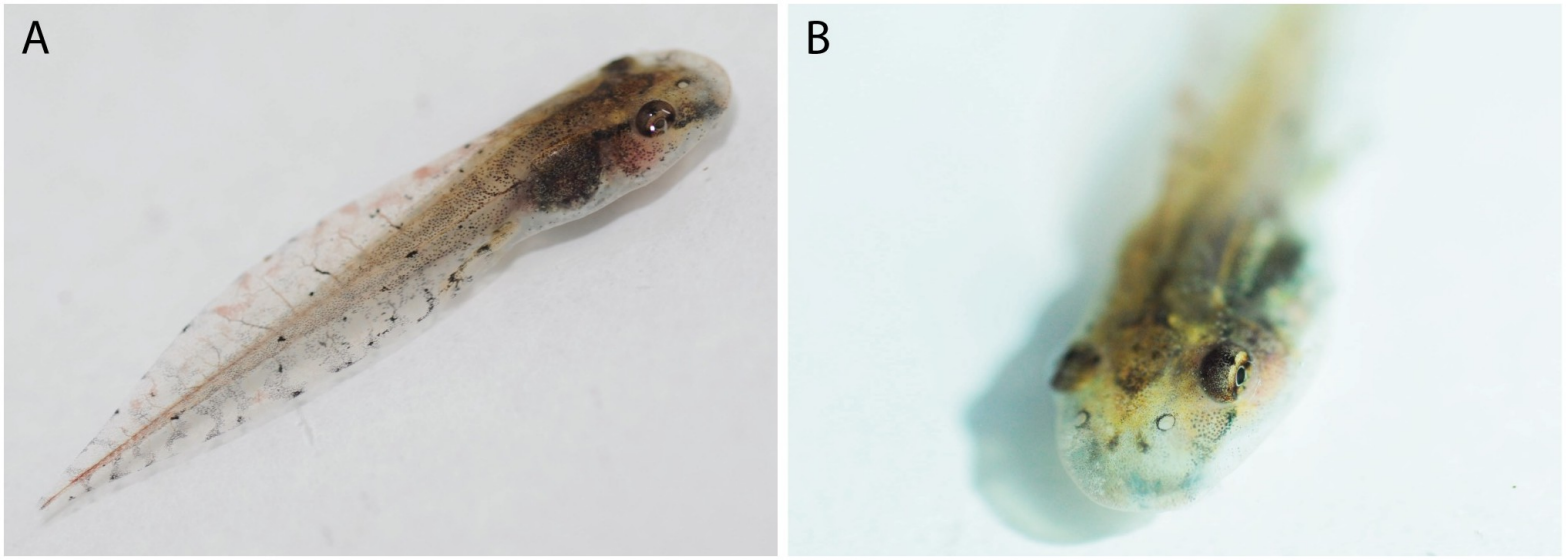
Coloration in life of a tadpole of *Scinax juruena* sp. nov. Lot ABAM 4101, Gosner stage 37.

In preservative (Fig 7), dorsum translucent grey; nostrils surrounded by dark grey pigmentation; dark grey diamond-shaped mark on interorbital region; dark grey canthal stripe from snout to anterior eye corner; dark grey stripe from posterior eye corner to body-tail insertion; dark grey blotch with frayed edges on infraocular region formed by very small dots. Ventral surface of body translucent grey, immaculate. Tail muscle brown with unpigmented blotches and black dots distributed irregularly; unpigmented, thin lateral line from body-tail insertion to tail. Fins translucent grey; light to dark grey, vertical vermiculation on both fins. Coloration in life (Fig 9) resembles that in preservative, except for an iris bronze with a dark brown horizontal bar and light orange blotches on dorsal fins; dorsum and tail muscle light brown.

### Comparisons with other species

Characters of compared species are presented inside parentheses unless indicated otherwise. One diagnostic character is presented for most compared species, except those whose morphology and advertisement calls are similar to the new species [7]. S1 Appendix contains data for additional specimens examined.

*Scinax juruena* sp. nov. differs from members of the *S*. *auratus*, *S*. *boesemani*, *S*. *danae*, *S*. *funereus*, *S*. *fuscovarius*, *S*. *granulatus*, *S*. *nasicus* and *S*. *rostratus* groups and *S*. *pachycrus* by the presence in adults of a bilobate vocal sac that reaches the level of the pectoral fold (vocal sac not reaching the level of the pectoral fold in all mentioned taxa: [5]); from members of the *S*. *squalirostris* group by its truncated snout in dorsal view (elongate acuminated in *S*. *squalirostris* group); and from members of the *S*. *fuscomarginatus* group by the absence of dorsolateral dark stripes (present in all species of the *S*. *fuscomarginatus* group; [23]).

*Scinax juruena* sp. nov. is distinguished from *S*. *manriquei* in having a bilobate vocal sac and the absence of a dorsolateral stripe (single vocal sac and light-colored dorsolateral stripe present: [70]).

*Scinax juruena* sp. nov. differs from *S*. *altae* by having a dorsum covered by dark brown spots and small blotches irregularly distributed, longitudinal stripe on thigh absent, an advertisement call with duration 189–227 ms and tadpoles with LKRF 2(2)/3 (dorsolateral and longitudinal stripes present on dorsum and thigh, respectively, call duration 140–180 ms, and tadpoles have LKRF 2(2)/3(1); [49]).

*Scinax juruena* sp. nov. is distinguished from *S*. *albertinae* by having a vocal sac with uniformly distributed dark melanophores, green bones and the dorsum covered by dark brown spots and irregularly distributed blotches (dark blotches restricted to the lateral region of the vocal sac, white bones, and a dark dorsolateral stripe; [7]). Moreover, *S*. *juruena* sp. nov. has an advertisement call with duration 189–227 ms, rate 68–89 calls/min, 30–35 pulses per note and dominant frequency 2,250–2,344 Hz (call duration 502–652 ms, call rate of 32–68 calls/min, 79–105 pulses per note and dominant frequency 3,811–4,543 Hz; Table 5). Tadpoles of *S*. *juruena* sp. nov. have the P-3 smaller than P-2 and P-1, and marginal papillae row formula (1)/3/1 (P-2 longer than P-1 and P-3, marginal papillae row formula (1)/2/1; [7]).

**Table 5:** Spectral and temporal parameters of the advertisement call of *Scinax juruena* sp. nov. and five closely related species.

	S. albertinae	S. cruentomma	S. exiguus	S. juruena sp. nov.	S. strussmannae	S. wandae
Reference	Ferrão et al. (2022)	Carvalho et al. (2015)	Carvalho et al. (2017)	Present study	Ferrão et al. (2018)	Ferrão et al. (2022)
Temperature (°C)	25	26.5–28.5	15–16	unknown	25	unknown
Calls analyzed	30	147	49	11	21	10
CD (ms)	559 ± 44 (502–652)	269 ± 22 (216–336)	1,148 ± 333 (632–1,638)	211 ± 15 (189–227)	106 ± 5 (97–115)	624 ± 17 (579–639)
ICI (ms)	590 ± 207 (342–1346)	582 ± 128 (325–1,281)	-	587 ± 58 (488–678)	338 ± 93 (221–601)	577 ± 115 (391–790)
CP (ms)	1,149 ± 210 (878–1,878)	-	-	796 ± 67 (677–88)	-	1,201 ± 121 (1,018–1,414)
CR (calls / minute)	54 ± 8 (32–68)	-	26 ± 8 (20–35)	76 ± 7 (68–89)	139 ± 24 (85–175)	50 ± 5 (42–59)
NP (pulses/call)	89 ± 7 (79–105)	47 ± 2.6 (39–54)	71 ± 8 (51–90)	33 ± 2 (30–35)	24 ± 6.6 (17–30)	88 ± 2 (82–90)
PD (ms)	3.4 ± 0.7 (2–6)	-	-	4.5 ± 0.5 (4–5)	2.1 ± 0.7 (2–3)	3.4 ± 0.6 (2–4)
IPI (ms)	2.9 ± 0.8 (2–7)	-	-	1.9 ± 0.4 (1–3)	2.1 ± 0.4 (1–3)	3.8 ± 0.6 (3–5)
PP (ms)	6.3 ± 0.6 (5–9)	-	-	6.5 ± 0.5 (6–7)	4.5 ± 0.5 (4–5)	7.2 ± 0.6 (6–8)
PRR (pulses/sec)	160 ± 14 (111–200)	175 ± 8 (154–193)	64 ± 14 (52–86)	156 ± 12 (143–167)	225 ± 25 (200–250)	139 ± 11 (125–167)
DF (Hz)	4,121 ± 149 (3,811–4,543)	4,654 ± 157 (4,406–4,875)	4,522 ± 290 (4,078–5,016)	2,318 ± 28 (2,250–2,344)	2,816 ± 93 (2,541–3,015)	5,097 ± 64 (4,952–5,146)

Measurement acronyms are defined in the text. Values depict average ± standard deviation (minimum–maximum).

*Scinax juruena* sp. nov. is distinguished from *S*. *blairi* by having males with SVL 24.1–27.6 mm, a bilobate vocal sac, calls composed of one pulsed note lasting 189–227 ms and containing 30–35 pulses, and a call rate of 68–89 calls per min (SVL 27.8–30.1 mm, single vocal sac, call duration 140–160 ms, 18–22 pulses per note and 102–133 calls per min [67]).

*Scinax juruena* sp. nov. differs from *S*. *cruentomma* by having a vocal sac covered with dark melanophores or blotches and a reddish-brown horizontal stripe on the iris (vocal sac immaculate and a red horizontal stripe on iris; [19, 68]). In addition, the call of *S*. *juruena* sp. nov. has an average duration of 211 ms ± 15 ms and 30–35 pulses/note (call duration 269 ms ± 22 and 39–54 pulses/note [73]). Finally, tadpoles of *S*. *juruena* sp. nov. have LKRF 2(2)/3, upper lip not emarginated laterally, MPRF (1)/3/1 and a dark brown horizontal bar on the iris (LKRF 2(2)/3(1), upper lip emarginated laterally, MPRF (1)/2/1 and a red horizontal bar on the iris; [68]).

*Scinax juruena* sp. nov. is distinguished from *S*. *exiguus* by having males with SVL 24.1–27.6 mm, a bilobate vocal sac, and the dorsum irregularly covered with dark brown spots and blotches (SVL 18.0–20.8 mm, single vocal sac, dark dorsolateral and lateral stripes; [69]). The advertisement call of *S*. *juruena* sp. nov. has a duration of 189–227 ms, 30–35 pulses per note and a dominant frequency of 2.250–2.344 Hz (call duration 632–1.638 ms, 51–90 pulses per minute and dominant frequency 4.078–5.016 Hz; [74]).

*Scinax juruena* sp. nov. differs from *S*. *karenanneae* and *S*. *lindsayi* by having a vocal sac covered with dark melanophores or blotches and a silver iris with a horizontal reddish-brown stripe (vocal sac without dark melanophores or blotches and no horizontal bar on the iris in both species; [17, 18]). Additionally, *S*. *juruena* sp. nov. has an advertisement call with duration 189–227 ms (call duration 80–100 ms in *S*. *lindsayi*; [17]).

*Scinax juruena* sp. nov. is distinguished from *S*. *staufferi* by having a dorsum covered with dark brown spots and small irregularly distributed blotches, calls with a pulse repetition rate of 143–167 pulses/s, and tadpoles have LKRF 2(2)/3 (irregular or continuous longitudinal marks, a pulse repetition rate of 100–130 pulses/s, and tadpoles have LKRF 2(2)/3(1); [49]).

*Scinax juruena* sp. nov. differs from *S*. *strussmannae* by having a larger body size, a vocal sac covered with dark melanophores or blotches, a reddish brown horizontal stripe on the iris and an advertisement call with a duration of 189–227 ms formed by 30–35 pulses/note (male SVL 20.2–22.5 mm, vocal sac immaculate, red horizontal stripe on the iris, and advertisement call with duration 97–115 ms and 23–27 pulses/note; [8]).

*Scinax juruena* sp. nov. is differentiated from *S*. *wandae* by having the dorsum covered with dark brown spots and irregularly distributed blotches, the snout truncate in dorsal view, and an advertisement call with a duration of 189–227 ms, 30–35 pulses/note and a dominant frequency of 2,250–2,344 Hz (four dorsolateral stripes, snout pointed in dorsal view, and call with duration 579–639 ms, 82–90 pulses/note and dominant frequency 4,952–5,146 Hz [7, 72]).

### Distribution and natural history

*Scinax juruena* sp. nov. inhabits dense ombrophilous forest on four islands in the archipelago of the Juruena River, southern Amazonia, northwestern Mato Grosso, Brazil. The forest is dominated by trees of the genera *Cecropia*, *Ochroma* and *Erythrina*, with *Heliconia* dominant in parts of the understory [75]. Massive copaiba (*Copaifera langsdorffii* Desf) and chestnut (*Bertholletia excelsa* Humb & Bonp) trees can be found on large islands (D. J. Rodrigues pers. comm.). Despite intensive sampling [76], we did not record any individual of *S*. *juruena* sp. nov. in forests on either bank of the Juruena River adjacent to the inhabited islands.

**Fig 10:**
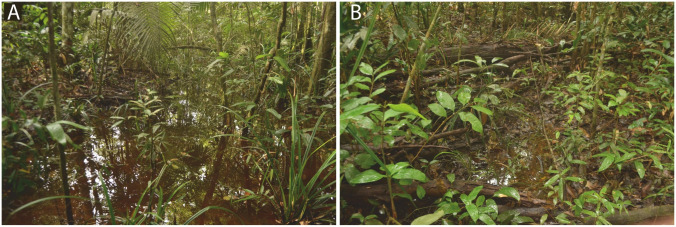
Breeding habitat of *Scinax juruena* sp. nov. on an island in the Juruena River, municipality of Cotriguaçu, state of Mato Grosso, Southern Amazonia, Brazil. (A) A large pond connected to the river. (B) A small pond formed by rainwater accumulation.

*Scinax juruena* sp. nov. breeds during the rainy season between November and May. Large choruses of breeding males were found after two or more days of consistently heavy rainfall. Males call while perched on shrubs and tiny trunks in or near large ponds formed by river flooding (Fig 10A) and small ponds formed by accumulation of rainwater in low-lying areas (Fig 10B). We found as many as three calling males sharing the same shrub. Other hylid frogs found breeding in the same ponds include *Osteocephalus taurinus* Steindachner, 1862, *O*. *leprieurii* (Duméril and Bibron, 1841) and *Dryaderces* sp. See [76]. for a checklist of species that inhabit the west bank of the Juruena River near the type locality of *S*. *juruena* sp. nov.

## Discussion

Although *Scinax juruena* and *S*. sp. 19 (sensu Araujo-Vieira et al. [5]) inhabit comparable riverine environments with similar flooding regimes, their distinct morphologies strongly support the hypothesis that these taxa represent distinct species. *Scinax juruena* differs from *S*. sp. 19 (Fig 4) by having the head as wide as long, a vocal sac covered by dark melanophores or blotches, and a well-defined horizontal reddish brown stripe on the iris (head longer than wide, no dark melanophores or blotches in males and an inconspicuous red horizontal stripe on the iris in *S*. sp. 19 from Madeira River; S2 Fig). *Scinax* sp. 19 occurs in the Madeira, Purus and Tocantins basins, while *S*. *juruena* is known only from the Juruena River (Tapajos Basin); their closest known localities are far apart (~ 700 km). Unsequenced specimens identified as *S*. (aff., cf.) *cruentomma* are reported from riverine environments in the Juruá, Negro, Tapajos, Solimões and Xingu rivers [73, 77–79]. Including samples from these localities in future taxonomic revisions will likely increase the number of species in the *S*. *cruentomma* group.

*Scinax blairi* was described by Fouquette and Pyburn [67], based mostly on individuals from near the confluence of the Guaviare and Ariari rivers in Colombia (approximate geocoordinates 2°33’N, 72°45’W). The acoustic similarity between *S*. *blairi* and *S*. *staufferi* was noted by Fouquette and Pyburn [67] and corroborated by Ferrão et al. [7], who also highlighted the acoustic similarity of these species and others assigned to the S. *cruentomma* group. Although molecular data are unavailable for topotypic *S*. *blairi*, we suggest that *S*. sp. (*S*. *cruentomma* clade 62 sensu Vacher et al. [14]; *S*. *wandae* sensu Guarnizo et al. [11]; *S*. cf. *wandae* sensu Araujo-Vieira et al. [5]) and *S*. *blairi* may be conspecific. Individuals of *S*. sp. were collected by Guarnizo et al. [11] near an affluent of the Ariari River ~140 km from the type locality of *S*. *blairi*. Additionally, the coloration of the type series resembles that of *S*. sp. (see BOLD:ABA0452 at the BOLD website; [80]). Molecular and morphologic analyses based on fresh material from the type locality of *S*. *blairi* might resolve this issue.

*Scinax juruena* is the first snouted treefrog to be described from northwestern Mato Grosso and the second one named from southern Amazonia [23]. Five other species of *Scinax* inhabit forests near the type locality: *S*. *fuscovarius*, *S*. *garbei*, *S*. *nasicus*, *S*. *nebulosus* and *S*. *ruber* [76]. However, recent phylogenetic studies conducted elsewhere in Amazonia demonstrate that these taxa represent species complexes and that remote areas in Amazonia likely harbor a large number of unnamed *Scinax* [10, 12, 14, 81].

Numerous small and large dams are planned for the Juruena Basin [32, 33], including both downstream and upstream from the islands in the Juruena River where *Scinax juruena* occurs. Fluvial islands are usually affected by dam construction, even when such projects do not form large lakes, and this is likely to be the case with islands in the Juruena River. For example, all islands in the portion of the Madeira River (Rondônia, Brazil) directly impacted by the lake of the Santo Antonio Power Plant have been totally or partially flooded. Disruption of seasonal water flows downstream from reservoirs is another important by-product of the construction of hydroelectric power plants [82] that directly impacts the biological cycle of water-dependent species [83].

*Scinax juruena* is currently known only from four fluvial islands in a section of the Juruena River with relatively well-preserved riverine forests and with an estimated extent of occurrence (EOO) of 0.54 km^2^. Although further inventories in other sections of the Juruena River are required to better estimate the species’ EOO and degree of endemism, the construction of numerous hydroelectric power plants in the region will likely jeopardize the species’ survival. Moreover, the region already suffers environmental impacts such as illegal mining, overfishing, unsustainable agriculture and uncontrolled logging. Pending additional data needed to properly assess its conservation status according to International Union for Conservation of Nature (IUCN) Red List Criteria, we recommend that *S*. *juruena* be categorized as Data Deficient (DD). However, if an EOO less than 5,000 km^2^ is confirmed, the species could be classified as Endangered (EN) based on criterion B1ab(iii) [84].

We strongly urge Brazilian licensing institutions to conduct environmental impact assessments that include comprehensive sampling of islands in the Juruena River and adjacent regions to evaluate the extinction risk of *Scinax juruena* and other organisms, and to implement meaningful conservation measures before the river is overwhelmed by hydropower projects.

## Supporting information

S1 Table Species, voucher identification numbers, collecting localities and GenBank accession numbers of DNA sequences used in this study. (XLSX)

S2 Table Morphometric measurements of adults of *Scinax juruena* sp. nov. (XLSX)

S3 Table Morphometric measurements of tadpoles of *Scinax juruena* sp. nov. (XLSX)

S1 Appendix Additional specimens examined. (DOCX)

S1 Fig Bayesian phylogenetic tree of *Scinax* based on a fragment of 16S rRNA. (TIF)

S2 Fig Dorsolateral and ventral views of *Scinax* sp. 19 from the west bank of the upper Madeira River, Rondônia, Brazil. (A–B) Adult male, APL 14930. (C–D) Adult female, APL 16897.
(TIF)
