# Supplementary material for: A new snouted treefrog (Anura, Hylidae, Scinax) from fluvial islands of the Juruena River, southern Brazilian Amazonia
Source: PLoS One. 2024 Jan 31;19(1):e0292441. doi: 10.1371/journal.pone.0292441 (PMC10830056; doi:10.1371/journal.pone.0292441)
Supplement: S1 Appendix — (DOCX) [file pone.0292441.s004.docx]

**S1 Appendix. Additional specimens examined.**

***Scinax albertinae***. BRAZIL: Amazonas, Novo Airão, Rio Negro Sustainable Development Reserve (INPA-H 42872 [holotype]; INPA-H 42863, 42857, 42861, 42867–69, 42874 [paratopotypes]; INPA-H 42856, 42858, 42862, 42864–66, 42870–71, 42875–76, 42873 [paratypes]). ***Scinax baumgardneri***. VENEZUELA: Amazonas: (MCZ-A 28563 [holotype]). ***Scinax blairi***. COLOMBIA: Vaupes, near junction of Rio Guaviare and Rio Ariari (MCZ-A 81819 [paratype]). ***Scinax boesemani***. SURINAME: Paramaribo: near Zanderij (RMNH 12601 [holotype, photo]), (MCZ-A 52833 [paratype]). BRAZIL: Roraima: Caracaraí, Viruá National Park (INPA-H 25972, 25974). ***Scinax chiquitanus***. BRAZIL: Rondônia: Porto Velho (INPA-H 35554–35558, 35560). ***Scinax cruentomma***. ECUADOR: Napo: Santa Cecilia (KU 126587 [holotype, photo]); Orellana: Parque Nacional Yasuní (QCAZ 8184), Río Napo (QCAZ 43772, 44754). BRAZIL: Amazonas: Careiro da Várzea, Ramal do Purupuru (INPA-H 34697). ***Scinax funereus***. ECUADOR: Orellana: Río Napo, Primavera (QCAZ 43799, photo), Tambococha (QCAZ 55280, 55283; photo); Napo: Limoncocha (MCZ-A 97672). ***Scinax fuscomarginatus***. BRAZIL: Roraima: Boa Vista, Maracá Ecological Station (INPA-H 34634, 34646, 34661, 34662); Caracaraí, Viruá National Park (INPA-H 19371, 19372, 19376, 19378, 19383, 19384). ***Scinax garbei***. BRAZIL: Roraima: Caracaraí, Viruá National Park (INPA-H 25964, 27496). ECUADOR: Napo: Limoncocha (MCZ-A 97672). ***Scinax madeirae***. BRAZIL: Rondônia: Alta Floresta, Corumbiaria Park (INPA-H 7050, 7051); Porto Velho (MCZ-A 64371 [paratype]). ***Scinax nebulosus***. BRAZIL: Pará: Alter do Chão (INPA-H 34647, 34653); Rondônia: Costa Marques, Real Forte Príncipe da Beira (INPA-H 34641); Roraima: Caracaraí, Parque Nacional do Viruá (INPA-H 27535–27537). ***Scinax onca***. BRAZIL: Amazonas: Berurí (INPA-H 20582, 20586, 34585 [paratypes]; INPA-H 34584 [holotype]; INPA-H 34581, 34583, 34587 [paratypes]); Rondônia: Porto Velho (INPA-H 34588–34595 [paratypes]). ***Scinax proboscideus***. BRAZIL: Amazonas: Manaus, Colosso Reserve at PDBFF (INPA-H 10304); Presidente Figueiredo, Vila Pitinga (INPA-H 1870); Pará: Oriximiná (INPA-H 304). ***Scinax ruberoculatus***. BRAZIL: Amazonas: Careiro da Várzea, BR-319, km 100 (INPA-H 34598, 34600, 34601, 34604, 34614, 34615, 34622, 34624, 34627, 34629 [paratypes]), km 168 (INPA-H 34602 [paratypes]); Borba, BR-319, km 220 (INPA-H 34610, 34620 [paratypes]); Beruri, BR-319, km 220 (INPA-H 34608 [paratypes]), km 360 (INPA-H 34599, 34607, 34609, 34611, 34612, 34617, 34618, 34621, 34625, 34626, 34628, 34630 [paratypes]); Manicoré, BR-319, km 400 (INPA-H 34603, 34606, 34616, 34623 [paratypes]); Tapauá, BR-319 km 450, Nascentes do Lago Jari National Park (INPA-H 34605, 34613, 34619 [paratypes]; INPA-H 34665 [holotype]). ***Scinax squalirostris***. URUGUAY: 15 km northeast of San Carlos, Alvarez Farm (MCZ-A 25761 [holotype of *Hyla evelynae*]). ***Scinax strussmannae***. BRAZIL: Amazonas: Tapauá, Nascentes do Lago Jari National Park (INPA-H 34688 [holotype]; INPA-H 34689–34692, 34700 [paratypes]).
