# Supplementary figures and images for: A new snouted treefrog (Anura, Hylidae, Scinax) from fluvial islands of the Juruena River, southern Brazilian Amazonia
Source: PLoS One. 2024 Jan 31;19(1):e0292441. doi: 10.1371/journal.pone.0292441 (PMC10830056; doi:10.1371/journal.pone.0292441)

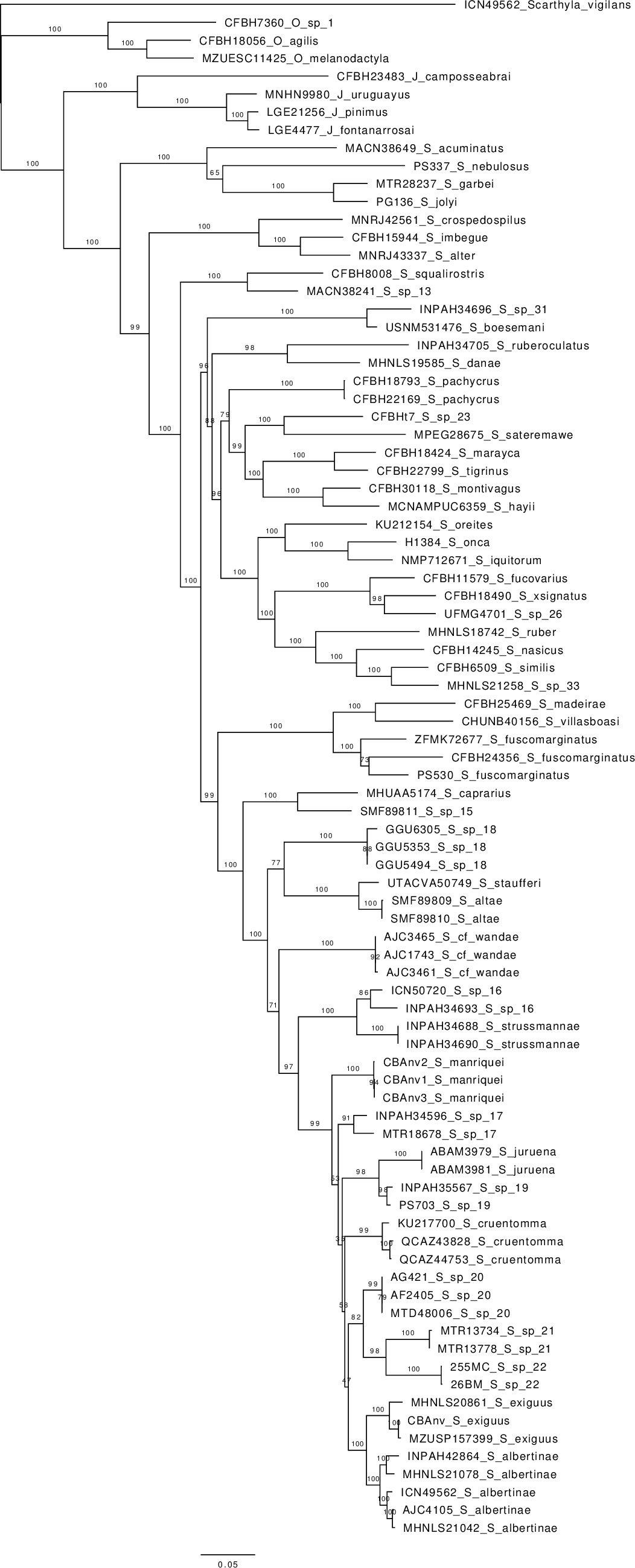

Supplement: S1 Fig — (TIF) [file pone.0292441.s005.tif]

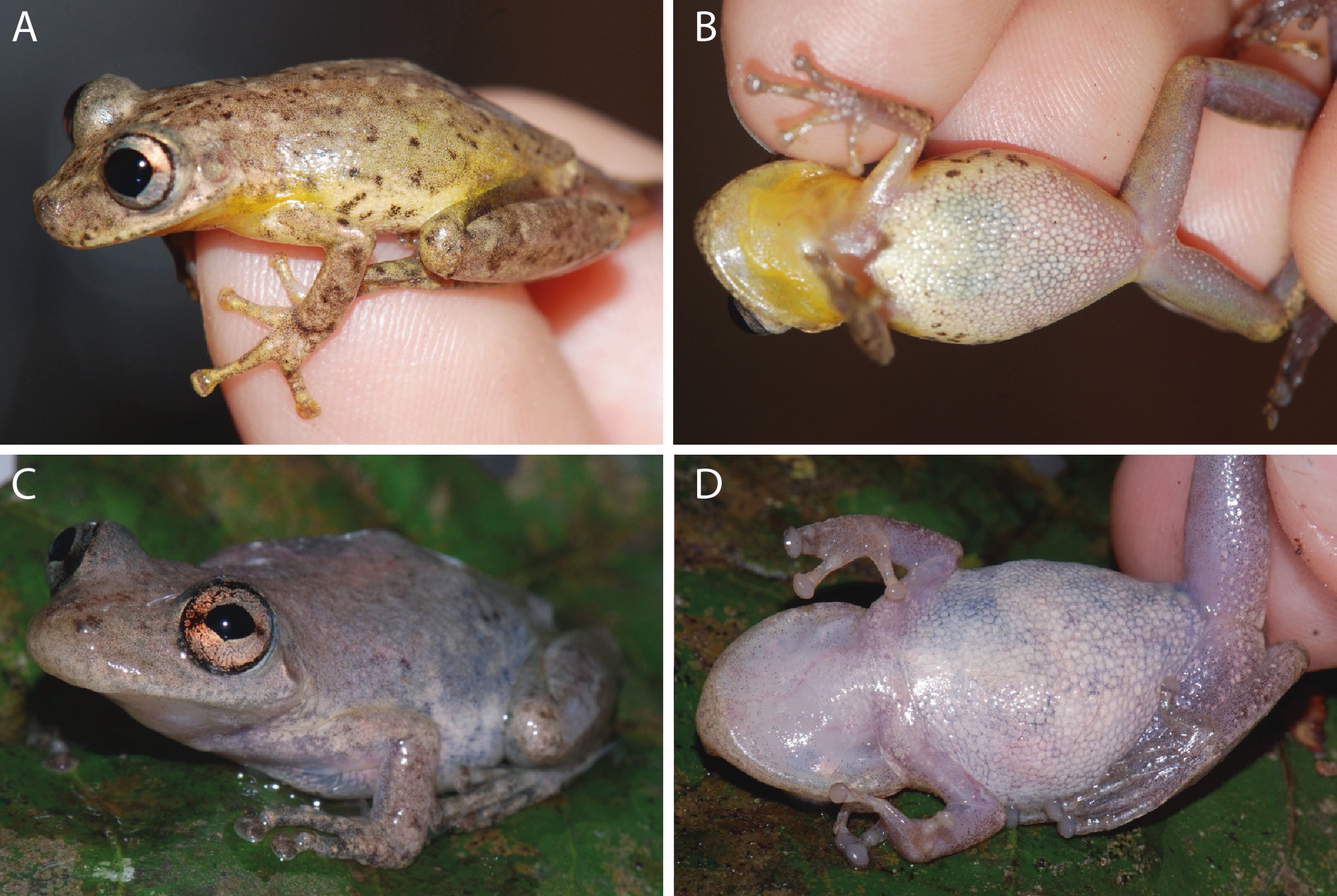

Supplement: S2 Fig — (A–B) Adult male, APL 14930. (C–D) Adult female, APL 16897. (TIF) [file pone.0292441.s006.tif]
